# Modern Approaches and Emerging Biological Therapies to Treat Fracture Nonunion

**DOI:** 10.3390/pharmaceutics17111457

**Published:** 2025-11-11

**Authors:** Julian Wier, Hannah Shelby, Sarah Bergren, Joseph T. Patterson, Jay R. Lieberman

**Affiliations:** 1 Department of Orthopaedic Surgery, Keck School of Medicine, University of Southern California, Los Angeles, CA 90033, USA; 2 Alfred E. Mann Department of Biomedical Engineering, Viterbi School of Engineering, University of Southern California, Los Angeles, CA 90033, USA

**Keywords:** nonunion, fracture healing, induced membrane, distraction osteogenesis, bone graft, BMP, orthobiologic therapy, gene therapy, scaffold, immunomodulation

## Abstract

Fracture nonunion remains an unresolved complication after extremity fracture, with notable costs to patient quality of life and health systems. Nonunion is defined by the inability of fracture ends to unite without evidence of progressive healing over time. Approximately 2 to 10% of all fractures go onto nonunion, with increased rates observed in specific fracture locations and patient populations. Despite advances in fixation techniques and bone grafting, current treatments remain limited and frequently fail to restore durable bone healing. In this review, the current state of emerging biologic and bioengineering therapies for nonunion will be summarized, with a focus on how these advances may shift treatment from palliative reconstruction toward durable healing. Biological therapies such as growth factors, stem cells, and gene-modified constructs show promise but face challenges of short half-life, inconsistent efficacy, and safety concerns. Emerging approaches, including controlled-release scaffolds, immunomodulatory materials, stem cell-derived exosomes, and gene therapy platforms, offer opportunities to more precisely restore the osteogenic, angiogenic, and immunologic environment required for union.

## 1. Introduction

Extremity fractures represent a common and morbid physical injury frequently seen after higher energy trauma. Globally, over 178 million new fractures were estimated in 2019 by the Global Burden of Diseases, Injuries, and Risk Factors Study group [1,2]. While the majority of fractures go on to heal, a notable subset may not. The incidence of nonunion widely varies, with reported rates ranging from 2% to 50%, depending on the fracture type and presence of concomitant risk factors [3,4,5,6]. The true incidence is likely between 2% and 5% for the majority of cases, though specific bones, such as the scaphoid, clavicle, and femur have a risk exceeding 10% [3,4,5,6,7,8]. Further, there are subsets of fractures and injury patterns which may portend a nonunion rate of well over 30% [9,10]. Fracture-related factors that influence nonunion risk include location, presence of concomitant fractures, open fractures, and poor residual fracture alignment after initial reduction attempts [4,11,12]. To that end, studies have reported close to a 20% nonunion rate in open tibial fractures, with the rate increasing if there is vascular compromise [12,13,14,15]. Due to the significant prevalence of nonunited fractures, understanding their causes and management strategies is of high importance.

Nonunion is defined by the United States Federal Drug Administration as a fracture which has not united by nine months after injury or one in which there is failure of progression towards union over three months [16]. While this definition can be useful to define a broad framework for understanding nonunion, patient and fracture specific factors must also be considered to more precisely capture the clinical picture. To more pragmatically characterize nonunion, others have defined it as a fracture that will not unite without further intervention [17]. Radiographic measures of nonunion have also been proposed. These include the Radiographic Union Scale in Tibia fractures (RUST) and the Radiographic Union Scale in Humeral fractures (RUSHU) scales, which score the probability of successful healing [18]. In addition to radiographic findings, laboratory measures are also key in defining the exact etiology of nonunion. These measures include the evaluation of inflammatory markers, such as C-reactive protein (CRP) to rule out infectious causes, as well as metabolically active endocrine factors, like vitamin D and parathyroid hormone [19]. Importantly, none of these measures should be used in isolation to define or predict the risk of nonunion, thus highlighting the patient-specific framework necessary to accurately diagnose this pathology.

Nonunion has been classified into several distinct categories, including septic and aseptic forms. Aseptic nonunion is further subclassified into hypertrophic, oligotrophic, and atrophic forms which references both the radiographic appearance of the fracture, as well as the presumed local microenvironment driving the observed radiographic findings [20]. A hypertrophic nonunion is presumed to have poor fixation, thus leading to excessive motion about the fracture ends, however, with good local biological healing potential [21]. This combination results in excessive callus formation, but without discrete resolution of fracture lines nor remodeling of the cartilaginous scaffold. Conversely, atrophic nonunion represents poor local biology which is radiographically characterized by lack of callus about the fracture site and resorption of the fracture ends [21]. While these two classifications are important conceptually, the majority of nonunions are best classified as oligotrophic nonunions which have a combination of poor fixation and poor local biology, and commonly appear with minimal callus [22]. These classifications help physicians define nonunion but its impact is better understood by examining the risk factors that lead to poor outcomes and the consequences on patients’ lives.

## 2. Clinical Context

### 2.1. Clinical Significance

Nonunited fractures have a significant impact on patients’ quality of life, often causing a notable loss of mobility and severe pain [23,24,25,26]. Several studies report substandard quality of life for patients with nonunion, comparable to multiple sclerosis and certain cancers [23,24,25,26]. Additionally, there is a significant impact on mental health. Patients with nonunion experience increased levels of anxiety and depression, related to their declining health, mobility issues, multiple procedures, uncertainty surrounding the healing process, and social isolation related to their persistent health challenges and prolonged treatment course [27,28,29,30]. Nonunited fractures also impose significant socioeconomic burdens on both the patient and the healthcare system as a whole. Direct costs, such as office visits, increased number of surgical procedures, and prescriptions increase expenses for the patient [31,32,33]. Additionally, patients require increased physical therapy sessions, home health services and prolonged hospital stays, further exacerbating their physical and financial burden [31,33,34]. Moreover, indirect costs, primarily related to lost productivity and wages from the inability to work, represent the largest financial cost of nonunions [26,27,31,32,35,36]. Given the extensive economic impact of nonunion through both direct and indirect costs to the patient, the healthcare system, and to society, the development of effective treatment solutions is vital.

### 2.2. Risk Factors

There are many risk factors for nonunion. Injury-related factors are particularly consequential, compromising the mechanical stability and local biological environment for healing. These are commonly observed in higher energy injury mechanisms and include soft tissue damage and greater bone loss leading to difficulties in maintaining fracture stability [4,6,11,27]. These factors are often associated with injury to the local vascular supply, which decreases the availability of oxygen and nutrients, thereby delaying healing and impairing an effective immune response. Given the risk of wound contamination during the injury, surgery, and postoperatively, the inability to mount an effective immune response may result in a fracture related infection, which further heightens nonunion risk [4,6,27]. Furthermore, several comorbid health conditions have been associated with nonunion, including diabetes, rheumatoid arthritis, and primary osteoarthritis which have been shown to increase the risk of nonunion by up to 40% [4,11,27]. Additionally, modifiable patient factors such as obesity, alcoholism, and smoking amplify this risk [4,14,27]. Smoking has been shown to impair fracture healing by reducing blood flow, in part through suppression of vascular endothelial growth factor (VEGF [VEGFA]) and von Willebrand factor (vWF [VFW]), which are essential for angiogenesis. Smoking also decreases the expression of genes critical for osteogenesis, including alkaline phosphatase (ALP [ALPL]) and bone morphogenetic proteins (BMPs) [37]. Similarly, alcohol disrupts the Wnt pathway, a molecular pathway important for bone healing and the genesis of osteoblasts [38]. Many of these risk factors are thought to influence bone healing by creating a pro-inflammatory environment that promotes osteoclasts and decreases osteoblastic activity, leading to increased bone resorption [22,39,40,41,42,43]. In particular, diseases such as osteoarthritis, diabetes and rheumatoid arthritis lead to chronic, systemic inflammation which negatively influences bone remodeling [39,41].

While comorbidity related risk factors for non-union have been established, genetic predisposition has also emerged as an area of investigation. In a study comparing peripheral venous blood samples from patients with nonunion to successfully healed fractures, polymorphisms within the platelet derived growth factor (PDGF) gene emerged as a potential genetic risk factor [44]. Additionally, a gain-of-function polymorphism within a matrix metalloproteinase, an enzyme critical to extracellular matrix remodeling, was associated with accelerated fracture healing [45,46]. Freedman et al. investigated single nucleotide polymorphisms (SNPs) in BMPs, reporting an inverse relationship with bone mineral density (BMD). Similarly, a genome-wide association study further identified ADAMTS18 and TGFBR3 as genes contributing to inter-ethnic variation in BMD [47]. Overall, these polymorphisms have several implications in the potential for early identification of patients at risk and targeted therapeutic strategies.

## 3. Pathophysiology

### 3.1. Bone Healing

**Figure 1:**
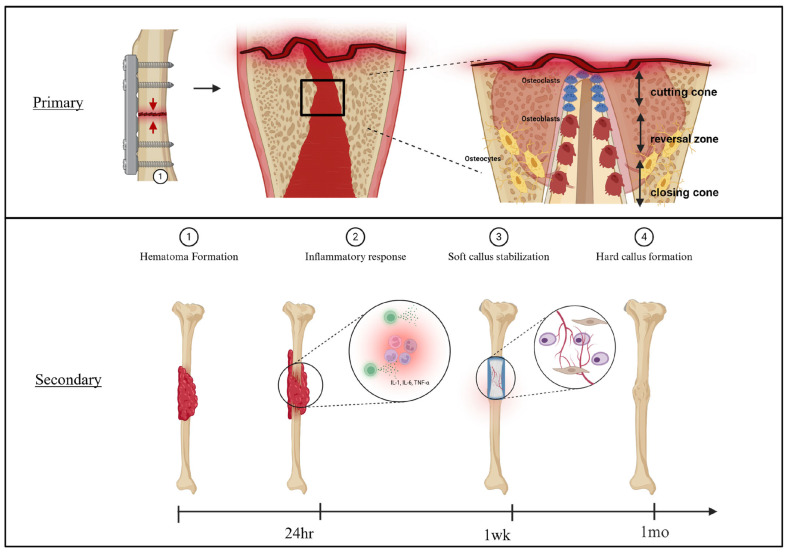
Schematic of primary and secondary bone healing. Primary bone healing (**top**) occurs under conditions of absolute stability, where osteoclasts create cutting cones that traverse the fracture line, followed by osteoblasts depositing lamellar bone in the reversal and closing zones, resulting in direct remodeling without callus formation [48]. Secondary bone healing (**bottom**) occurs under conditions of relative stability and follows a staged progression beginning with (1) hematoma formation within 24 h, providing a preliminary scaffold for inflammatory cell infiltration, (2) an inflammatory phase marked by neutrophil and macrophage recruitment and release of cytokines, (3) stabilization of a cartilaginous soft callus with early vascular invasion and progenitor cell differentiation over the first week, and (4) formation of a mineralized hard callus by one month, ultimately leading to bridging and consolidation of the fracture [49]. Created in BioRender. Shelby, H. (2025) https://BioRender.com/l9yx33f.

Bone healing can occur through primary (direct) or secondary (indirect) healing (Figure 1).

Primary healing occurs when the fragments are reduced and stabilized under compression with minimal motion at the fracture site. Perren’s strain theory lays the foundation for this understanding. Strain is the measure of the relative deformation of a material when a given force is applied and it ultimately determines the type of tissue that can form in the fracture gap. For instance, in the condition of absolute stability with inter-fragmentary strain less than 2%, lamellar bone formation will occur without significant callus formation (primary healing). Conversely, a range of 2–10% will encourage secondary bone healing with callus formation. Strain outside this range disrupts osteogenesis, promotes fibrocartilaginous differentiation of mesenchymal stem cells (MSCs), and ultimately increases the risk of delayed union or nonunion [50]. In high-strain environments, woven bone cannot tolerate the deformation, thus interrupting the hard callus phase, preventing stable bridging.

Expanding on primary bone healing, in the context of absolute stability, bones are able to heal via remodeling of the lamellar bone and Haversian canals with minimal callus formation. This process involves osteoclasts, specialized cells responsible for bone resorption, forming “cutting cones” at the end of the Haversian canals near the fracture site. The cutting cones create tunnels across the fracture lines, followed by osteoblasts generating bone across the plane [48]. Paradoxically, the process of direct bone healing typically takes longer compared to indirect healing. It is worth noting that if rigid fixation is imperfect, potentially through micro-motion from poor compression or implant loosening, the strain may exceed tolerance for direct bone formation, and the callus will not form [51]. This will ultimately lead to the development of a nonunion. At the cellular level, when mechanical stability is achieved and interfragmentary strain remains low, MSCs experience a stiff microenvironment that enhances cytoskeletal tension, activating integrin–FAK–Src complexes and downstream mitogen-activated protein kinase/extracellular signal-regulated kinase (MAPK/ERK) cascades. These pathways converge on Wnt/β-catenin signaling, promoting nuclear translocation of RUNX2 and Osterix (SP7) to drive osteogenic differentiation [52,53]. Conversely, excessive micromotion or unstable fixation alters these mechanical inputs, leading to diversion toward chondrogenic or fibroblastic phenotypes with impaired bone formation [54].

**Figure 2:**
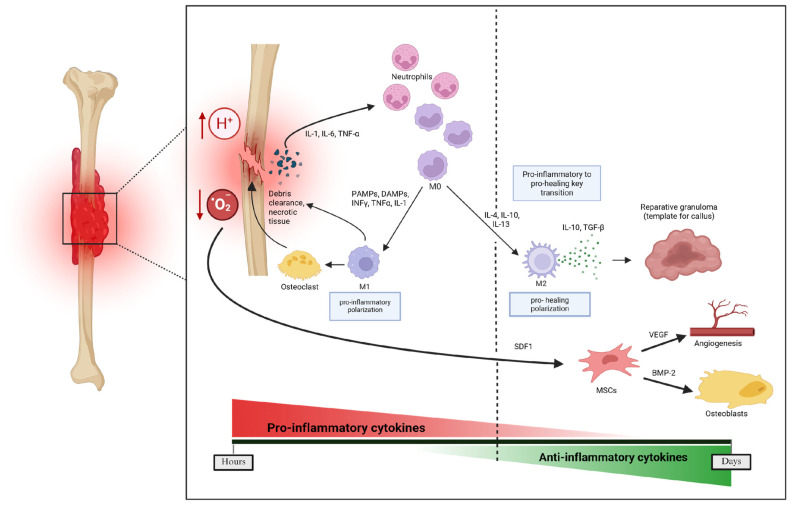
Cellular and molecular events during the early inflammatory phase of secondary bone healing. Following fracture, hypoxia, acidosis, and tissue necrosis trigger the release of debris and inflammatory mediators. Neutrophils are rapidly recruited and secrete pro-inflammatory cytokines (interleukins [IL-1, IL-6], tumor necrosis factor- α [TNF-α]), while monocytes (M0) differentiate into M1 macrophages under the influence of pathogen-associated molecular patterns (PAMPs), damage-associated molecular patterns (DAMPs), and inflammatory cytokines (interferon-γ [INF-γ], TNF-α, IL-1) [55,56]. M1 macrophages promote osteoclast activity for necrotic tissue clearance and sustain a pro-inflammatory environment [57]. Polarization of M1 macrophages to M2 macrophages is driven by IL-4, IL-10, and IL-13, marking the resolution of early inflammation to initiate tissue repair via secretion of IL-10 and transforming growth factor-β (TGF-β) [58]. M2 macrophages stimulate reparative granuloma formation, serving as a template for callus. Concurrently, stromal cell-derived factor 1 (SDF1 [CXCL12]) recruits MSCs, which differentiate into osteoblasts (via BMP-2 signaling) and support angiogenesis (via VEGF signaling) [56]. Created in BioRender. Wier, J. (2025) https://BioRender.com/7ezf2ps.

Secondary bone healing is more common and involves both intramembranous and endochondral ossification. Following injury, disruption of blood vessels supplying the bone leads to the formation of a hematoma, which serves as a scaffold for immune and progenitor cell infiltration (Figure 2).

This acute inflammatory phase represents the first stage of secondary bone healing and generally peaks within 24 h and ends after 7 days. It is characterized by wide platelet degradation that results in the subsequent release of pro-inflammatory cytokines, including interleukins (IL-1, IL-6) and TNF- α [59,60]. These cytokines result in the recruitment and proliferation of macrophages and neutrophils. First, neutrophils are recruited and then monocytes/macrophages infiltrate into the fracture site. PAMPs and DAMPs, as well as pro-inflammatory cytokines (e.g., INF-γ, TNF-α, IL-1) promote the M1 (pro-inflammatory) phenotype of macrophages. M1 macrophages amplify the inflammatory reaction, recruit additional immune cells, and phagocytose microorganisms, necrotic tissue, and the provisional fibrin matrix. After neutrophils and macrophages clear the area of cellular debris, the process transitions to the resolution of inflammation and formation of a reparative granuloma that forms the template for the subsequent callus. During the resolution of acute inflammation, macrophages are polarized from an M1 phenotype to an M2 (pro-healing) phenotype by anti-inflammatory cytokines IL-4, IL-10, and IL-13. (Figure 2) Bone marrow derived stem cells (BMSC) are attracted locally by cytokines such as TNF-α and SDF1 [39,61]. Recruited inflammatory cells and BMSCs participate in critical inter-cellular communication or crosstalk via pro-inflammatory cytokines and anti-inflammatory cytokines. Moreover, the release of multiple local proteins, including BMPs and additional growth factors (e.g., VEGF, PDGF, fibroblast growth factor-2 [FGF-2], TGF-β) further potentiates the initiation of osteogenesis and angiogenesis [62]. The following phase is characterized by the formation of a callus, which involves both intramembranous ossification and endochondral ossification. At the periosteum, intramembranous ossification occurs and a hard callus forms directly, whereby periosteal MSCs differentiate into osteoblasts that directly form woven bone [56,59].

**Figure 3:**
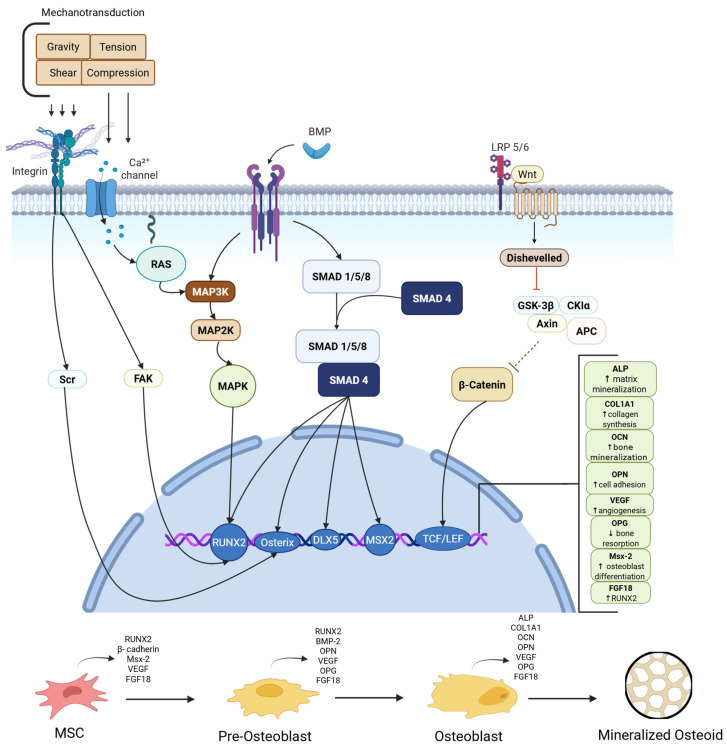
Cellular mechanisms underlying osteogenesis. Bone morphogenetic protein (BMP) signaling activates SMAD1/5/8, which associate with SMAD4 and translocates to the nucleus, while canonical Wnt/β-catenin signaling, mediated through LRP5/6 and Dishevelled, stabilizes β-catenin to promote transcriptional activity [63,64]. In parallel, mechanotransduction activate integrins and Ca^2+^ channels, initiating intracellular signaling cascades through Src, FAK, and MAPK pathways [52]. These converging pathways regulate key transcription factors, including RUNX2, Osterix, DLX5, MSX2, and TCF/LEF, which drive the expression of osteogenic genes such as ALP, collagen type I (COL1A1), osteocalcin (OCN [BGLAP]), and osteopontin (OPN [SPP1]) [52,65]. Together, these mechanisms coordinate matrix mineralization, angiogenesis, and osteoblast differentiation. The lower panel illustrates the stepwise progression from MSCs to pre-osteoblasts, mature osteoblasts, and ultimately mineralized osteoid, highlighting the integrated molecular and cellular processes essential for bone formation. Created in BioRender. Bergren, S. (2025) https://BioRender.com/qt1yy6z.

BMPs, along with other regulatory factors, play an integral role in this process of callus formation (Figure 3).

BMP initiates the signal transduction cascade by binding to cell surface receptors and forming a heterotetrameric complex composed of two dimers of type I and type II serine/threonine kinase receptors. Activated BMP receptors phosphorylate Smad proteins (specifically Smad 1, Smad 5, and Smad 8) and these bind to a common mediator protein, Smad 4, to form a complex [66]. This complex translocates to the nucleus where it functions as a transcription factor. Downstream, the complex cooperates with additional transcription factors (e.g., RUNX2, Osterix, DLX5, MSX2) to upregulate key genes promoting osteogenesis. These include ALP, involved in matrix mineralization, COL1A1, integral to type I collagen synthesis, OCN, a late osteoblast marker, OPN, involved in bone matrix adhesion, and VEGF, which promotes angiogenesis for callus vascularization [67]. Smad-independent pathways also contribute to bone healing. These include MAPK cascades and small GTPases, which promote osteoblast differentiation and early proliferation of osteoprogenitor cells. RUNX2, known as the “master regulator of osteoblast differentiation,” also inhibits chondrocytes from acquiring the phenotypes of permanent cartilage chondrocytes, allowing this cartilage to be replaced by bone, making RUNX2 a key player in endochondral ossification [68]. Finally, the last stage of bone remodeling can persist for several months. The process involves the coordinated interaction of signaling pathways, featuring BMP, FGF, and parathyroid hormone-related protein (PTHrP). Continued migration of osteoblast and osteoclasts are contributors to “coupled remodeling”, the dynamic equilibrium between bone resorption and bone formation [69].

### 3.2. Impaired Healing

Despite this coordinated and tightly regulated process, bone healing does not always occur successfully. Among areas of investigation, the acute inflammatory phase of bone healing is one component that seems to influence the progression of nonunion. Imbalance in the early macrophage polarization signals and other paracrine factors which promote the orchestrated transition through each phase of healing can lead to delays and potential cessation in osteogenic progression. Persistence of an M1-dominant milieu has been linked to nonunion, as well as impaired callus formation [70,71,72]. Conversely, the coordinated induction of M2 macrophages promotes vascular invasion and osteoblast recruitment [73,74]. Additional murine models have been used to demonstrate that unregulated acute inflammation impairs fracture healing [75,76,77]. In a clinically relevant model, Recknagel et al. used a blast wave generator to induce chest trauma following an osteotomy, demonstrating delayed healing with increased neutrophil infiltration and elevated pro-inflammatory cytokines at the fracture site [76,77]. Moreover, unstable fractures which are commonly associated with delayed healing, have been shown to exhibit significantly more cytotoxic T cells and leukocytes in the hematoma neighboring the injury when compared to those with stabilized fractures [78,79].

The local biology, including the role of adequate blood supply, is another key factor implicated in non-union. It was long theorized that atrophic non-unions were avascular and biologically inert. However, this framework has been largely disproven, with evidence that these tissues retain vascularity even in the setting of atrophic nonunion. To that end, Reed et al. reported no significant difference in vessel density between atrophic non-unions, hypertrophic nonunions and healing fractures [80]. Nevertheless, adequate blood supply remains a key component in promoting healing and reducing the risk of fracture related infections. Local protein factors, such as BMPs have also been implicated in the dysregulation of bone repair. Kloen et al. reported evidence of ongoing BMP signaling in non-union tissue, while other studies have revealed an imbalance between BMP’s expression and that of their inhibitors in this setting [81,82,83]. Similarly, matrix metalloproteinases (MMPs) have been highlighted in non-union pathophysiology as these proteins bind and degrade BMP-2 [84]. The molecular and biochemical pathways underlying impaired fracture repair remain an area of investigation, with ongoing research working to identify therapeutic targets to restore osteogenesis.

## 4. Current Solutions to Address Nonunion

Several important factors are necessary for bony union, which include the presence of osteogenic cells, osteoinductive stimulus, osteoconductive scaffold, and a robust vascular supply [85,86]. Current interventions are designed to reintroduce these key elements of osteogenesis that are deficient in nonunited fractures. Techniques such as bone grafting, local cellular and molecular therapies, or a manufactured scaffold, aim to mimic the bone growth environment and compensate for any deficient elements needed for bone formation [85,87,88] (Table 1). These interventions strive to augment missing structural or biologic elements needed for durable fracture healing and mimic the original pathways necessary for bone development and repair.

### 4.1. Nonsurgical

Non-invasive methods are more commonly considered in cases of delayed union or in patients where surgical intervention is precluded. These include prolonged immobilization and external modes of bone growth stimulation (BGS). Initial management of delayed union can consist of an extended period of bracing or casting of the affected limb [16]. However, these methods commonly do not result in complete fracture healing and carry a much higher risk of malunion which may further exacerbate the functional deficits caused by the fracture. BGS is an adjunct to immobilization, with the goal of reducing the rate of nonunion and facilitating healing if delayed. Examples of external BGS include low intensity pulsed ultrasound (LIPUS), electrical stimulation (ESTIM)/pulsed electromagnetic fields (PEMF), and extracorporeal shockwave therapy (ESWT). The clinical interest in LIPUS has grown over time, while ESTIM has fallen out of favor, with several reviews demonstrating unfavorable healing rates ranging from 55 to 61% [105,106]. Conversely, the use of LIPUS in the setting of nonunion has been shown to have an 82% healing rate, with even greater healing rates for fragility fracture patients with non-unions [107,108]. Despite this encouraging data, several randomized controlled trials evaluating the effect of LIPUS on nonunion healing demonstrated no superiority compared to a control group [109,110]. As such, there is limited adoption of these external BGS methods in modern orthopaedics.

### 4.2. Surgical Solutions

#### 4.2.1. Traditional Surgical Approaches

**Figure 4:**
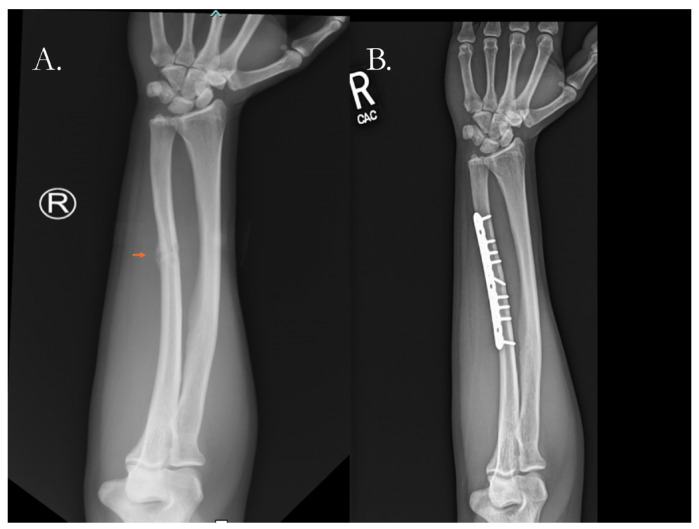
Ulnar nonunion with compression plating. 35-year-old female that presented 2 months after sustaining an ulnar fracture and was found to have nonunion of her fracture. The patient was treated with an open reduction and internal fixation with a compression plate. (**A**) Radiograph from when the patient was first diagnosed with nonunion (orange arrow). (**B**) Radiograph 3 months after fixation.

Nonunited fractures are commonly treated with internal fixation using plates or intramedullary nails (IMNs), typically combined with a bone graft [111,112,113]. These require either increasing or decreasing the strain the fracture sees depending on the type of fixation primarily used and the character of the resulting nonunion. In the setting of hypertrophic nonunions, which commonly have excessive bone formation and impede appropriate bony opposition, partial decortication is often employed to reduce the osseous burden and allow for optimal fixation [112]. Different surgical constructs are used to elicit primary or secondary healing. Implants like IMNs, which promote secondary healing, allow for the mechanical continuity of bone to be restored so that daily movements can physically stimulate healing through mechanotransduction [82]. In certain cases, compression plating, which evokes primary healing, is used, in which a metal plate is fixed to the fracture segments and screws are used to provide a compressive force between the bony ends (Figure 4).

**Figure 5:**
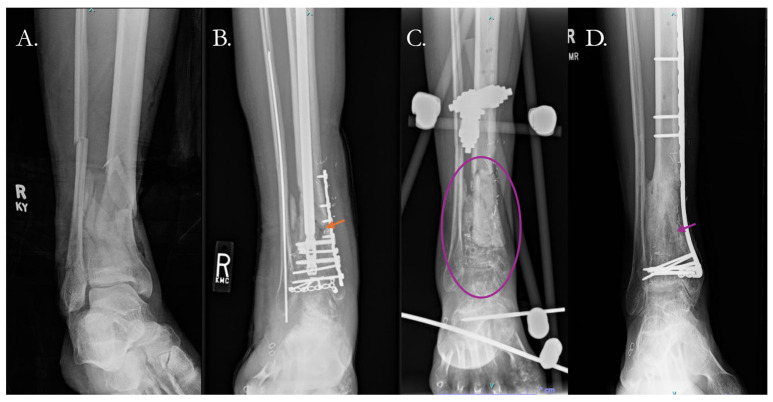
Nonunion of pilon fracture with induced membrane and local biologic therapy. 27-year-old male who was in a motor vehicle accident resulting in a pilon fracture. One year after his injury, the patient was diagnosed with septic tibial nonunion and underwent repair with an induced membrane, iliac autograft and rhBMP-2. (**A**) Radiograph of initial injury demonstrating comminuted distal tibial fracture with intra-articular extension and ipsilateral fibula fracture. (**B**) Radiograph of primary hardware at over 6 months after initial fixation when the patient was diagnosed with infected nonunion. Orange arrow pointing at persistent fracture lines. (**C**) Radiograph of antibiotic spacer (purple oval) resulting in an induced membrane (not visible) with external fixator in place to hold alignment. (**D**) Radiograph 3 years following repair with evidence of fracture consolidation (purple arrow) and new plate fixation.

Additional techniques in the setting of large bone defects include the Masquelet technique and distraction osteogenesis. The Masquelet technique involves implanting a polymethyl methacrylate cement spacer in the bone defect that acts as a foreign body to induce a vascularized membrane [114]. The cement spacer induces inflammation and edema at the site of nonunion causing a membrane to form. This membrane will release growth factors and cytokines to induce angiogenesis, forming a vascular supply for the healing bone [115,116]. This process can take several months, after which the spacer is removed and the empty void is filled with autologous bone graft (Figure 5).

Gaillard et al. demonstrated that this technique can be successful in humeral fracture nonunions, with 15 out of 15 nonunion achieving union [117]. Success has also been shown with nonunions of the tibia, radius, ulna and the femur, with rates of union between approximately 85–95% [65,118,119,120,121,122].

**Figure 6:**
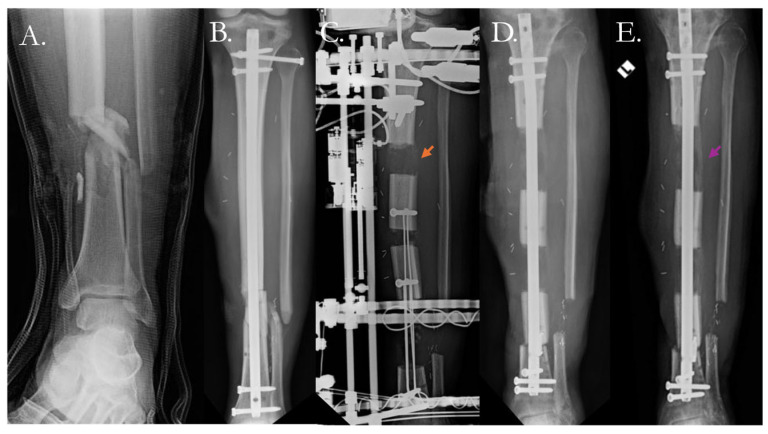
Distraction osteogenesis of the tibia following nonunion. 57-year-old male with an open tibial and fibular septic nonunion following a motorcycle collision. (**A**) Radiograph of initial injury demonstrating a displaced segmental distal third tibia fracture with ipsilateral fibula fracture. (**B**) Initial fracture fixation using intramedullary nailing to allow for secondary bone healing. (**C**) Radiograph after debridement and saucerization of regions of tibial osteomyelitis with dynamic external fixator in place. Region of regenerate highlighted with orange arrow. (**D**) Definitive fixation with intramedullary nail after removal of dynamic external fixator at 4 months after initial external fixator placement. (**E**) Most recent radiograph at 6 weeks after intramedullary nail placement with evidence of early callus about region of regenerate (purple arrow).

Distraction osteogenesis has shown promise in promoting healing in nonunited fractures with large defects [123]. In this technique, an osteotomy is performed, and the bone is progressively distracted as new bone fills in the gap (Figure 6).

Additional strategies include an externally controlled dynamic intramedullary nail that uses a magnetic mechanism to provide compression or distraction at the fracture site. In a study of five tibial and nine femur nonunions, an intramedullary compression nail was able to achieve union in 13/14 cases [124]. While these surgical techniques can be successful, the biological or mechanical environments may not always be fully restored. Moreover, these techniques may require multiple surgical procedures which increase the risk of complications, including infection and devascularization of the local environment, which further prolongs healing and increases nonunion risk. In this setting of multiple surgeries and potential complications, an immense psychosocial burden is placed on patients and patients are commonly confronted with having to choose amputation despite multiple prolonged treatment courses [30]. As such, opportunities to reduce the nonunion risk during the primary surgery or limit the interventions to a single repeat surgery are of high importance.

#### 4.2.2. Surgical Solutions with Biologic Therapy

In addition to targeting the biomechanical environment, surgical exposure allows for the direct application of biologic therapies. Bone grafting is a commonly used option to bridge small bone defects and can be sourced autologously or as an acellular allograft [125,126]. Autologous bone graft has several advantages over allograft, which only serves as an osteoconductive matrix, as it contains a patient’s own osteogenic cells and osteoinductive proteins, as well as serving as a scaffold for osteoconduction. Common regions used to obtain autograft include the iliac crest, the tibia, and the femoral intramedullary canal [125]. However, local graft can be obtained from nearly any site. The major limitation of autologous bone graft is availability and donor site morbidity, as it is typically harvested from a discrete site outside the zone of injury thus requiring additional surgical exposure. Vascularized bone graft is an attractive option for large bone defects as it not only provides the benefits of autologous bone, but also a direct vascular conduit to the implanted tissue. However, the risk of morbidity and additional surgical procedures is particularly relevant for vascularized bone graft, such as free fibula transfers, which require significant surgical expertise and are associated with clinically relevant rates of wound complications and chronic pain [127].

While autograft is limited to ~20–40 cm3 of available tissue, bone allograft is widely available at large quantities, however, at a significant monetary cost [128]. Allogeneic bone grafts are harvested from one individual, living or cadaveric, and implanted into another. These grafts are predominantly osteoconductive in nature, serving as a scaffold for new bone formation but may maintain some osteoinductive properties [129]. Critically, once prepared and sterilized, these grafts no longer contain osteogenic cells or key growth factors, serving primarily as a scaffold for bony ingrowth [126]. The use of allografts has limited utility in nonunion treatment because of their poor biological activity with evidence of higher risk of re-fracture and nonunion when used in large bone defects [126]. Despite the widespread use of these bone grafts, the aforementioned limitations highlight the need to investigate adjunctive therapies.

**Figure 7:**
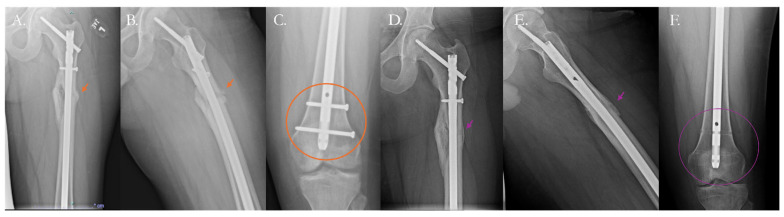
Femur nonunion with BMAC and nail dynamization. 27-year-old male with a nonunion following intramedullary nailing of a displaced comminuted femur fracture. Six months after his initial surgery the patient was diagnosed with nonunion of the fracture. (**A**,**B**) Radiographic findings of femur nonunion with residual fracture line through region of hypertrophic callus (orange arrow). (**C**) Distal locking screws (orange circle) in initial intramedullary nail construct. (**D**,**E**) Radiographic evidence of union at one year after nail dynamization and BMAC placement, with resolution of multicortical fracture lines (purple arrow). (**F**) Evidence of removed distal locking screws (purple circle) to dynamize intramedullary nail for compression of proximal fracture segment.

Bone marrow aspirate concentrate (BMAC) can be used as the sole graft material or as a potential adjunct to grafts for bone regeneration (Figure 7).

BMAC is obtained through aspiration of bone marrow, usually from the iliac crest. The aspirate is then processed to concentrate MSCs and associated growth factors such as VEGF, PDGF, and BMPs [130,131,132]. BMAC has shown to lead to union in 75% to 100% of cases, particularly with septic nonunions [93,133,134]. Further, the addition of BMAC to the bone grafts was shown to accelerate healing times in nonunion compared to patients treated with standard surgical fixation and bone graft alone [89]. Despite its promising clinical applications, one of the main limitations is the variable amount of stem cells and the heterogeneity of its contents across different individuals. In a study of 8 patients, the percentage of MSCs within BMAC generated from each patient’s bone marrow ranged from 0.0001% to 0.003% [135]. Another study highlighted how the age of a patient and the origin of the bone marrow influenced the amount of mononuclear cells, which includes stem cells, within BMAC, with younger patients having a higher proportion of monocular cells and the iliac crest producing more cells than the tibia [136]. Furthermore, another disadvantage of this method is the iatrogenic pain and morbidity associated with bone marrow retrieval. Overall, BMAC is a promising adjunct for nonunion treatment, but improved isolation techniques and more high-quality research are needed to better understand its efficacy in this context.

Platelet rich plasma (PRP) is another adjunct to bone grafts that can be used in nonunion cases. PRP is typically sourced by isolating platelets and their associated growth factors from autologous blood. Through this process, PRP is proposed to deliver a concentrated milieu of critical growth factors, such as PDGF, TGF-β, and VEGF, as well as immunomodulatory protein such as interleukins which promote bone healing [94,137]. Wang et al. demonstrated that in a retrospective study of 66 patients with femoral nonunions, PRP significantly increased healing rates by 20% and decreased healing time by almost 4 months [138]. Additionally, similar to BMAC, the use of PRP has demonstrated enhanced healing rates relative to surgical fixation and bone grafts [89]. Although PRP has the potential to be effective in improving healing in nonunion, the evidence remains mixed. A recent meta-analysis showed that PRP may lead to a faster time to union but results were inconclusive on whether it positively influenced healing rates [94]. Specifically, concerns remain about the reproducibility of PRP, with evidence that the molecular composition varies widely between individuals and collection methods [139,140,141]. Given these inconsistencies, more data is required to precisely define its effect on nonunion and its optimal use.

## 5. Emerging Strategies for Nonunion Repair

Emerging strategies leverage local biology to enhance bone formation through the use of growth factors, immunomodulation, scaffolds, local drug delivery, stem cells and gene therapy. Each of these components targets a key factor in bone stimulation (Table 2).

### 5.1. Exogenous Growth Factors

Growth factors target the upregulation of osteogenic differentiation and proliferation, with potential targets including not only osteoinductive factors, but also those related to angiogenesis and immunomodulation [168,169]. Multiple families of growth factors are presently being investigated as potential therapeutic targets, such as VEGF, PDGF, TNF-ɑ, and the TGF-β superfamily including BMPs [168] (Table 3). However, their utility is limited by rapid diffusion and short local retention times, which hinder sustained biological activity at the target site [170]. As a result, localized delivery systems are being developed to enhance the spatial and temporal availability of these factors, thereby promoting more effective therapeutic responses.

#### 5.1.1. BMP

Although several strategies leveraging osteoinductive growth factors have also shown promise, BMP-2 has emerged as the most popular to enhance bone regeneration. Recombinant BMP-2 (rhBMP-2) is an analog designed to stimulate differentiation of MSCs into osteoblasts which has achieved FDA approval as an adjunctive treatment modality for acute open tibial fractures [144]. A series of preclinical studies have demonstrated that rhBMP-2 enhances bone healing when combined with various delivery systems and scaffold materials. It has been shown that rhBMP-2 administered with human periosteum-derived cells recapitulate physiological fracture healing in mouse tibia, while subsequent studies in rat models confirm rhBMP-2′s role in promoting hypertrophic chondrocyte formation and mineralized bone bridging [183,184]. When used with allografts or synthetic scaffolds, rhBMP-2 significantly improved integration and mechanical strength, often outperforming autograft controls. To that end, several studies demonstrated that rhBMP-2-loaded constructs induce rapid lamellar bone formation and bridging in rat and canine models [185,186,187]. These combined approaches highlight emerging strategies to augment rhBMP-2 functionality by potentiating its delivery and enhancing bone stability which could ultimately be applied to nonunion scenarios. Despite some evidence of clinical efficacy, concerns related to local adverse tissue reactions due to the high initial dose required to achieve prolonged effects have hampered its widespread use. Specifically, its short half-life necessitates a high local dose to sustain therapeutic effects [188]. The delivery system is a central limitation to its use, with the only FDA approved carrier for rhBMP being an absorbable clinical sponge [189,190]. While the sponge allows localized delivery, it exhibits poor control over release kinetics and can lead to rapid burst delivery. This necessitates using a supraphysiologic dose to maintain therapeutic levels. The high burst release has been associated with excessive soft-tissue swelling and formation of seromas [191,192]. Additionally, the high-dose use of rhBMP-2 has been associated with heterotopic bone formation, soft tissue swelling, osteoclast-driven bone resorption, and oncologic risks in doses exceeding 40 milligrams [193]. These adverse effects underscore a need for an improved delivery system with controlled-released scaffolds. Multiple approaches have been proposed to achieve this controlled release, including the use of scaffold encapsulation or more specific affinity-based methods targeting key interactions between BMP-2 and functionalized surface substrates. A highly cited example is heparin-mediated BMP-2 delivery using heparin microparticles blended into carriers [184,194]. In a rat critically sized femoral defect, this approach increased in vivo BMP-2 retention, tightened spatial localization of new bone to the defect, and reduced heterotopic ossification [184]. A complementary line of studies engineered heparan-sulfate variants (HS3) embedded in collagen/mineral composites to triple BMP-2 retention and extend release to ~4 weeks, yielding significantly greater osteogenic differentiation in vitro [195]. Similarly, heparin-dopamine conjugates demonstrated significantly improved bone formation than heparin alone on a biologic scaffold [196]. Collagen-anchoring bridge proteins with dual affinity for BMP-2 and collagen have also produced strong, site-restricted osteogenesis in murine spinal fusion models at reduced doses by tethering BMP-2 directly to the matrix [197]. These data show that specific molecular interactions are able to modulate spatiotemporal release profiles to enhance defect repair while curbing off-target bone formation.

Beyond optimizing release profiles, research involving BMP has shifted toward more clinically relevant chronic nonunion models. These models use delayed treatment following a period of nonunion development, better simulating clinical scenarios. In this context, Bosemark et al. identified a combination of BMP-7, and systemic bisphosphonates as most effective in nonunited rat femurs [198,199]. Similar findings were found by DeBaun et al. and Kaipel et al., who used variations of BMP-2 delivery via polycaprolactone scaffolds and fibrin matrices to achieve complete union in chronic defects [99,200]. However, limitations to BMP-2 therapy have also been noted in this setting, such as dose-dependent heterotopic ossification, and inferior mechanical properties in BMP-induced bone formation [201,202]. Other adverse effects noted by these studies included ectopic bone formation, rapid BMP clearance, adipogenesis, and bone cysts. These findings underscore the therapeutic potential of BMPs in nonunion repair while highlighting the need for careful dose modulation and safety evaluation prior to clinical translation.

#### 5.1.2. Alternative Molecular Mediators of Bone Healing

In addition to BMP, several alternative growth factors have been explored for the treatment of nonunion, with varying degrees of success [203]. VEGF enhances angiogenesis and has shown promise in bone regeneration when delivered via gene-activated matrices, although its efficacy falls short when compared to BMP [204]. Murine models have demonstrated that supra-physiologic concentrations of VEGF can accelerate healing of long bone fractures; however, evidence of direct benefit in human fracture healing remains limited [200,205]. Additionally, PDGF has been considered a “starter” signal for the wound-healing cascade in tissue regeneration. However, while studies on supplemental rhPDGF have shown improvements in bone healing, it generally fails to achieve complete union alone [206]. Combinations of rhPDGF with β-TCP and collagen matrices have shown encouraging results in stimulating union; however, additional data on its efficacy are presently needed [200,207,208,209,210]. Other investigations of molecular targets include sclerostin inhibitors such as romosozumab, which have demonstrated significantly improved bone volume in ulnar defects of cynomolgus monkeys [104]. Another endogenously derived growth factor which has shown promise in promoting bony union is parathyroid hormone (PTH). By upregulating RUNX2 and OSX, PTH promotes osteoblast differentiation, an important osteoinductive process in bone repair [211]. Pulsed administration of rhPTH, specifically teriparatide, has been shown in preclinical animal models to enhance bone healing by stimulating callus formation, improving mechanical strength, accelerating endochondral ossification, and increasing union rates [212,213,214,215]. From a clinical perspective, several studies demonstrate a modest benefit in rhPTH administration in fracture healing. A meta-analysis of eight randomized trials involving 524 patients found that rhPTH treatment significantly reduced healing time, improved pain relief and functional outcomes, albeit without affecting overall union rates [216]. Data in younger, non-osteoporotic patients is even less clear, with only a single trial of 13 patients examining the effect of rhPTH on union [217]. Although these mediators of bone healing have potential, there is collectively limited data in their utility and efficacy in treating fracture nonunion.

### 5.2. Immunomodulation

Another evolving strategy in the treatment of nonunion is targeting the acute inflammatory phase of bone healing, optimizing early progression toward bony union. Prostaglandin E2 (PGE2) is a proposed target in this inflammatory pathway that has shown promise in improving bone formation. Selective agonists of the PGE2 and PGE4 receptors have resulted in enhanced bone formation and biomechanical strength [218,219,220]. Furthermore, dual administration of a selective PGE4 receptor agonist and rhBMP-2 has demonstrated significantly improved bone healing in murine models [221]. By targeting a similar biological pathway, Rundle et al. demonstrated accelerated fracture healing in a rat model by overexpressing COX-2 using a retroviral vector system [222]. Biologic scaffolds are another proposed solution to facilitate the coordinated transition between these inflammatory phases and have shown promise in promoting regeneration. These are manufactured to ensure an optimal surface topography and an exact internal porous architecture to facilitate osteogenesis with maintained structural properties. Notably, these scaffolds have also been shown to modulate the immune microenvironment by enhancing immune cell adhesion, migration, and macrophage polarization [223]. By leveraging varied scaffold materials and properties, several studies have demonstrated increased M2 polarization and promotion of the restorative inflammatory phase [224,225,226,227,228]. Xuan et al. demonstrated that a 3D-printed ordered bredigite scaffold promoted M2 polarization by enhancing IL-10 expression while suppressing TNF-α, while also promoting BMSC proliferation, thereby accelerating defect healing in a rat critical-sized femoral model [224]. Incorporating both inflammatory and osteoinductive mediators has also demonstrated success in facilitating improved osteogenesis. Similarly, Xiong et al. reported that digital light processing-fabricated hydroxyapatite (HA) scaffolds with hierarchical porous structures regulated the immune microenvironment by increasing CD206^+^ M2 macrophages and reducing iNOS^+^ M1 macrophages, resulting in elevated BMP-2 secretion, VEGF release, and significantly improved bone formation in their rabbit skull defect model [227]. Others have engineered functionalized scaffolds using metal ions, bioactive molecules, or piezoelectric materials to provide specific osteoinductive cues, while also modulating macrophage phenotypes [223]. Another therapeutic approach involves the incorporation of BMP-2 and osteoimmunomodulatory agents, which have demonstrated significantly improved BMP-2-induced in vivo osteogenesis, suggesting that inflammatory inhibition is crucial for maximizing BMP-2 efficacy [229,230]. Given the essential role of the acute inflammatory phase in modifying the risk of nonunion, identifying how to optimally leverage growth factors and maximize scaffold design to promote bone regeneration will remain a key avenue of future research.

### 5.3. Scaffolds

Numerous in vitro and in vivo studies have evaluated the therapeutic performance of a broad range of scaffold compositions and architectures, particularly focusing on the capacity of these constructs to support vascularization and osteogenesis. These scaffolds widely range in terms of their material composition and therapeutic effect, with high degrees of modifiability through the use of bioengineering and 3D printing modalities (Table 4).

#### 5.3.1. Bioceramics

Synthetic bone substitutes can be used to support local osteoconduction with potential osteoinductive properties, as modifications to their surface topography can promote osteoblastic differentiation of MSCs by mimicking the mechanotransductive characteristics of the native extracellular matrix [245]. Bioceramics are a class of inorganic materials that have been widely explored for the treatment of long bone nonunion and segmental defects due to their innate bioactivity, structural rigidity, and ability to emulate the mineral phase of natural bone. They are composed primarily of calcium-phosphate ceramics such as HA and tricalcium phosphate (TCP), as well as bioactive glass (BAG). Additionally, bioceramics are able to be coated with synthetic biodegradable polymers such as polylactic acid (PLA), polyglycolic acid (PGA), poly-ε-caprolactone (PCL), or polylactic acid co-glycolic acid (PLGA), or natural compounds such as collagen and gelatin to improve structural strength [246,247]. For example, collagen-wrapped HA scaffolds implanted in rabbit femoral defects resulted in bone formation with mechanical strength equivalent to autologous graft and 3D printed PLA scaffolds delivering rhBMP-2 to sheep metatarsal defects significantly higher bone formation than controls [102,246]. Importantly, these materials can also be manufactured to fabricate anatomically customized scaffolds with specific porosity and mechanical properties [248,249]. Engineered microporous structures of bioceramics can facilitate angiogenesis and mineral deposition, with porous TCP scaffolds demonstrating greater bone volume and increased blood vessels in critical sized defect models [250,251]. Lu et al. reported that diamond pore TCP scaffolds induced osteogenic differentiation and significantly improved bone healing through the Ras pathway, implicating the link between mechanotransductory mechanisms and cell fate [252]. Several other studies have demonstrated success with piezoelectrically engineered barium titanate scaffolds to accelerate bone repair [253,254,255]. Conceptually these leverage load-driven piezoelectric currents to simulate the natural bioelectricity formed during native bone regeneration. Despite their promise, the majority of bioceramics remain brittle and mechanically inferior to certain synthetic polymers, and their regenerative efficacy may be more influenced by scaffold architecture or fabrication method than the ceramic composition itself [97,256]. Nevertheless, their integration with other polymers continues to expand their clinical potential in nonunion repair, with hybrid systems enabling controlled therapeutic delivery.

#### 5.3.2. Natural Polymers

Natural polymers have garnered substantial interest due to their intrinsic biocompatibility given their resemblance to native extracellular matrix components [257]. Materials currently under active investigation include gelatin, collagen, alginate, chitosan, silk fibroin, and hyaluronic acid, each exhibiting distinct degradation kinetics and fabrication compatibility. However, the biological origin of these materials also introduces variability in mechanical integrity and immune compatibility. Some formulations have been associated with increased inflammatory responses which may impair bone healing or elicit adverse host reactions [258,259]. Additionally, the mechanical strength of most natural polymers is inferior to synthetic alternatives, limiting their application in load-bearing environments unless reinforced or modified. To overcome these limitations, hybrid approaches incorporating natural polymers with ceramics, synthetic polymers, or growth factors have been explored to optimize scaffold mechanical integrity, degradation profile, and osteoinductive capacity in the treatment of bone nonunion [155].

Among natural polymers, gelatin has been widely utilized due to its derivation from collagen, excellent biocompatibility, and ease of fabrication into porous scaffolds. Gelatin scaffolds have been used as carriers for endothelial progenitor cells to enhance angiogenesis and bone regeneration in critically sized rat femur defects, demonstrating improved healing when compared to PRP [260]. Collagen, another prominent scaffold material, represents the primary organic component of bone, and its low immunogenicity and cell-adhesive properties make it ideal for tissue engineering applications [261,262]. When combined with calcium phosphates or demineralized bone matrix, collagen scaffolds can mimic the composite structure and mineral content of native bone. Schwarz et al. demonstrated significant bone regeneration in rat femur defect models using collagen sponges loaded with BMP-2, though the osteogenic response was largely attributed to the growth factor rather than the collagen matrix alone [263].

Other natural polymers under active investigation include alginate and silk fibroin. Alginate can be readily cross-linked under mild conditions to form hydrogels with adaptable porosity and mechanical stiffness. Although alginate is non-toxic and cost-effective, it lacks natural degradability in mammals and often requires irradiation or chemical oxidation to optimize its degradation kinetics [264,265]. Modified alginate scaffolds have been shown to sustain BMP-2 release and promote bone formation more effectively than unmodified versions or even collagen sponges in rat critical-sized defects [266,267]. Silk fibroin, derived from silkworm cocoons, exhibits favorable mechanical properties, slow biodegradation, and biocompatibility. These characteristics make it suitable for fabricating scaffolds with customized porosity and topography to support osteoconduction and vascular infiltration [268,269]. In a rat femoral defect model, Deininger et al. created anisotropically porous silk fibroin scaffolds coated with apatite and loaded with low-dose rhBMP-2 (2.5 µg), achieving substantial bone regeneration while reducing required growth factor concentration [270]. While no single natural polymer is universally optimal, strategic combinations of materials with bioactive agents can yield scaffolds that effectively support bone healing and nonunion resolution across a range of anatomical and mechanical contexts. However, these scaffolds will need to be tested in clinically relevant animal models to assess their efficacy in more biologically equivalent environments.

#### 5.3.3. Hydrogels

Hydrogels are garnering increased attention in the field of orthopaedics and bone regeneration. Due to the unique architecture of hydrogels, physiologic cues within the local environment (e.g., pH, temperature, etc.) allow them to degrade in response to stimuli, thus more effectively controlling the release of a therapeutic substance [271,272,273]. They can be made with a variety of organic substances such as chitosan, collagen, alginate, and gelatin to mimic the natural extracellular matrix [274]. Moreover, hydrogel mechanical properties have also shown to directly influence MSC fate, with biomimetic compositions promoting cell adhesion, osteogenic differentiation, and bone repair [275,276,277]. As such, hydrogels can also provide an environment for both osteogenesis and angiogenesis, further facilitating their effectiveness in promoting bone union [278]. Broadly, hydrogels have shown success as a medium for local drug delivery, with several in vitro and in vivo studies demonstrating improved healing and increased osteoinductivity when applying hydrogels together with local therapy [279,280,281,282,283]. To that end, hydrogels containing BMSCs or encapsulated rhBMP-2 implanted into bone defect models resulted in improved osteogenesis and successful union [277,284]. Furthermore, Yu et al. reported successful use of gelatin methacryloyl hydrogel scaffolds in conjunction with preosteoblast-derived matrices in a rabbit model of radial bone defects, highlighting their application in bone regeneration strategies requiring both bioactivity and structural support [279,280,281,282]. Even with their current successful applications in nonunion, hydrogels lack mechanical strength and are subject to rapid breakdown, particularly limiting their use in large bone defect scenarios [285,286,287]. Collectively, these studies underscore the versatility of hydrogels as biomimetic scaffolds that not only enable controlled therapeutic delivery but also provide a supportive microenvironment for osteogenesis and angiogenesis, thereby positioning them as promising adjuncts in bone regeneration and union; however, more studies are needed to discover mechanisms to limit the degradation and improve the mechanical strength of hydrogels without compromising their biocompatibility.

### 5.4. Stem Cell-Based Therapy

Stem cells represent a promising therapeutic avenue for bone union because of their capacity to differentiate directly into osteoblasts [101,149,288,289]. Among these, MSCs have garnered particular attention, given their ability to differentiate into osteoblasts, chondrocytes, and adipocytes while also exerting immunomodulatory and pro-angiogenic effects [290]. BMSCs remain the most extensively studied, although their harvest can be invasive and associated with donor-site pain [291]. Alternative sources include adipose-derived MSCs (ADSCs) and those harvested from synovial fluid, dental pulp, and umbilical cord blood, which have demonstrated comparable differentiation capacity to BMSCs [292,293,294,295]. Prior investigations using direct autologous MSC injections into fracture or nonunion sites have reported radiographic bridging and improved biomechanical properties within months of treatment [296,297,298]. In a recent meta-analysis of 21 studies including 866 patients, Cui et al. evaluated MSC therapy in the setting of nonunion, reporting improved early healing and lower rates of complication than with standard bone graft treatments [158]. Importantly, the regenerative efficacy of MSC therapy appears to depend on the differentiation status of transplanted cells. In a rat femoral nonunion model with periosteal cauterization and marrow ablation, percutaneous delivery of osteogenically predifferentiated MSCs (OCPs) significantly enhanced bone healing compared to undifferentiated MSCs and untreated controls [299]. ADSCs are increasingly studied as these cells can be readily obtained from procedures such as liposuction [300]. In a large animal model, Schubert et al. evaluated osteoblast-differentiated ADSCs in pigs with CT-confirmed fibrotic nonunion and treatment resulted in complete bone fusion, further supporting the therapeutic potential of stem cells in chronic nonunion repair [301]. However, overall union rates at final follow-up were equivalent to untreated cohorts. Taken together, MSC-based therapy holds substantial promise for accelerating fracture healing and nonunion repair. Ultimately, despite the regenerative potential of MSC therapy, the transplanted cells often show limited survival and a rapid decline in osteogenic activity within the defect site [302].

Scaffold-assisted MSC delivery using ceramic or polymeric constructs further enhances osteoconductivity and bone regeneration, with injectable hydrogels and decellularized matrices mimicking the biomechanical and biochemical milieu of native bone [303,304,305,306]. Studies by Maiti et al. and Ninu et al. demonstrated that scaffolds seeded with autologous, allogeneic, or xenogeneic MSCs accelerated healing of critically sized rabbit radial defects, with BMSCs enhancing bone formation particularly when combined with osteoconductive bioceramics such as silica-coated HA [307,308]. Similarly, nanohydroxyapatite/gelatin scaffolds pre-seeded with BMSCs and loaded with BMP-6 significantly enhanced in vitro osteogenesis and in vivo calvarial defect healing in rats [309]. Moreover, Kim et al. explored the osteogenic potential of canine ADSCs delivered via an osteogenic cell sheet seeded on a composite PCL/β-TCP scaffold in a canine radial segmental defect model which significantly outperformed undifferentiated ADSCs or scaffold-only controls in terms of new bone volume, highlighting the value of stem cells and scaffold pairing in large animal models of nonunion [310].

Exosomes, a subtype of extracellular vesicles (EVs), are thought to play a large role in the regenerative properties of stem cells and can be composed of lipids, proteins, mRNA or miRNA [311,312]. They are able to reach target cells either through diffusion to surrounding tissue or in circulation, using specific cell-mediated interactions through ligands or endocytosis, to release their contents into cells [312]. Several studies have investigated extracellular vesicle-based therapies, particularly the application of exosomes, in promoting fracture healing. Qin et al. demonstrated that BMSC-derived EVs suspended in a hydrogel enhanced osteogenic gene expression and osteogenic differentiation, enabling bone regeneration in a critical-sized calvarial defect rat model [313]. Further, Narayanan et al. cultured human marrow-derived stromal cells (HMSCs) with pro-osteogenic exosomes and observed that these exosomes were endocytosed by HMSCs and created an upregulation of key osteogenic growth factors such as TGF-*β*-1 and BMP-9 [314]. Narayanan et al. also performed an in vivo study using a murine model that showed collagen scaffolds containing HMSCs and osteogenic miRNA-based exosomes had increased vascularization and osteogenic differentiation compared to controls [314]. Moreover, exosomes offer a variety of advantages: the lipid bilayer protects its contents from degradation, they are thought to retain the immune privilege nature of their parent cells and they can be stored for long periods of time without loss of potency [311]. These studies demonstrate how stem cell derived exosomes can induce fracture healing while avoiding challenges of other therapies.

However, despite promising preclinical data, clinical application of stem cells still raises concerns. While MSCs are typically characterized by low immunogenicity, immune rejection remains a possibility with allogeneic use [315]. In a systematic review of 555 patients, approximately 15% of the patients treated with allogeneic MSCs developed associated antibodies [316]. However, these antibodies were found to not be correlated with treatment safety or cause significant side effects in patients. More critically, the long-term persistence and differentiation behavior of MSCs remain poorly understood, with theoretical risks of uncontrolled growth or tumorigenesis. Corcoran et al. showed MSCs can facilitate the spread of cancer cells into bone marrow, increasing the metastasis of cancer [317]. Though MSCs are not intrinsically oncogenic, they can facilitate tumor progression under certain conditions, making rigorous safety evaluations essential prior to widespread clinical adoption [318].

### 5.5. Integration of Gene Therapy

Gene therapy approaches involve the modification of a cell’s genetic material to induce differential expression of effector genes. In vivo and ex vivo viral vector or nonviral mediated systems have been used with varied efficacy, with ex vivo viral vector delivery serving as the gold standard at present [319,320,321]. Local MSC-based gene therapy presents an evolving paradigm for enhancing fracture healing, particularly in complex defects or dysvascular bone environments. By genetically modifying MSCs to overexpress specific therapeutic proteins, sustained and localized bioactivity can be achieved without the need for repeated protein administration. Viral vector-mediated gene delivery has further accelerated the translational potential of MSC-based therapies for bone healing. Adenoviral (AD), lentiviral (LV), and adeno-associated viral (AAV) vectors have all been used to transduce growth factor genes such as BMP-2, BMP-6, BMP-7, and VEGF, enabling both in vitro and in vivo application to fracture and fusion models [322,323,324,325]. This strategy has been successfully performed in animal models using several cell sources, including BMSCs and ADSCs [149,261,326,327]. In an early study, Lieberman et al. showed that BMSCs transduced with BMP-2 via AD vectors healed over 90% of critical sized femoral defects in a murine model with restored mechanical properties [149]. Further, Liao et al. showed that the co-expression of BMP-6 and VEGF in nude rates resulted in increased bone and blood vessel growth [261]. Kumar et al. also demonstrated that murine MSCs transduced to express with both BMP2 and VEGF led to greater bone formation and vascularity in a segmental bone defect model [326]. More recently, single-cell RNA sequencing of rat femoral defects treated with ex vivo regional gene therapy versus rhBMP-2 revealed that gene therapy not only induced a more robust chondrogenic response but also created an anti-inflammatory microenvironment with reduced fibroblast-driven profibrotic signaling, offering mechanistic insight into its advantages over recombinant protein delivery [328]. While ex vivo transduction is popular, it can be time consuming and requires isolation of patient compatible cells [329]. Given this, Baltzer et al. explored the efficacy of in vivo transduction by adding AD vectors encoding BMP-2 directly to rabbit femoral defects, resulting in significantly increased radiographic healing and stronger biomechanical properties of new bone [330]. Additionally, to shorten turnaround time, Evans et al. demonstrated a same-day gene therapy approach for bone defect repair by transducing fat and muscle tissue containing MSCs with AD-BMP-2 intraoperatively, then reimplanting it into critical sized rat femoral defects. This approach achieved bone bridging within 10 days and full mechanical restoration by 8 weeks, importantly demonstrating the effectiveness of rapid transduction [331]. Virk et al. took a similar approach harvesting femoral and tibial BMSC from Lewis rats, transducing the cells ex vivo with LV-TSTA-BMP-2, then reimplanting the transduced cells into a critical-sized femoral defect all within the same day, resulting in earlier radiographic healing and higher bone volume compared to the traditional two-step transduction approach [332]. Bougioukli et al. took this approach further, using a “next day approach” where human- derived BMSCs were isolated and transduced overnight to be implanted into critical-sized rat femoral defects the next day; however this approach did not lead to superior healing compared to the two-step approach [333]. While BMP is most often used, other gene constructs, including VEGF/BMP-4 and PDGF, have also proven effective in specific bone defect models, although efficacy may be ratio- and site-dependent [334,335,336]. Specifically, ratios of 0.2:1 and 1:1 VEGF transduced cells to BMP-4 transduced cells showed increased bone area in murine fracture models, with significantly increased bony bridging and union rates compared to a ratio of 5:1 or VEGF alone [336]. While many studies have looked at gene therapy for bone regeneration in rat models, Pelled et al. was one of the first to extend its application to a large animal model. Using a minipig, they demonstrated that fibrin gel cellularized with BMSCs expressing BMP-6 led to greater vertebral bone repair on microCT compared to their fibrin only control [96]. These investigators also evaluated various ways of delivering target genes into these stem cells without the use of viral vectors, such as with ultrasound and with nucleofection [96,323,324]. These studies underscore the therapeutic potential of viral vector–modified MSCs in orthopedic applications as well as the complexities associated with identifying optimal delivery modes.

An important limitation of these models is the pro-inflammatory response to viral vectors, as the immune system recognizes these vectors as infectious viruses, prompting inflammation and tissue infiltration of immune cells [337,338,339,340]. For example, stem cells transduced with an AD vector to express BMP-2 can lead to an inflammatory response that hinders bone formation [341]. Further, immune suppression with FK506 has been shown to lead to superior bony bridging in rat femoral critical-sized defects treated with AD-BMP-2 transduced MSCs [342]. Despite the immunogenicity of viral vectors, ex vivo gene transfer has been used to significantly limit the in vivo exposure to viral antigens, thereby reducing the immune response to these vectors [149,343,344]. Vakhshori et al. implanted human ADSCs transduced with LV-BMP-2 into a critical-sized defect of athymic rats, showing a majority of defects healed at 12 weeks, matching the performance of rhBMP-2 while demonstrating superior histology and biomechanics over control groups [344]. Moreover, there is evidence of limited biodistribution and toxicity related to LV gene therapy. For instance, Bell et al. demonstrated minimal viral copies in internal organs 84 days after implantation of human ADSCs transduced with a lentivirus to express BMP-2 into a rat femoral defect model [343]. In contrast to AD or AAV systems, LVs produce a more limited innate immune response. LVs have been found to express fewer viral proteins post-transduction and support effective transgene expression with less inflammatory signaling [340]. Comparing bone repair studies, LV-BMP2 featured more robust bone formation with months-long expression, whereas AD methods exhibited shorter expression periods with stronger host response [345,346,347]. To that end, Feeley et al. found that in two murine bone-formation models, LV delivery sustained transgene expression for nearly 3 months versus approximately 21 days with AD systems [346]. Although further research is needed to understand the safety and optimal delivery of gene therapy in bone regeneration, the integration of gene therapy with stem cell therapy demonstrates great potential for the treatment of nonunions.

## 6. Future Directions

With the development of novel technologies in the space of bioengineering and molecular therapies, emerging techniques and approaches show early promise in furthering bone regeneration and nonunion repair. These include the use of nanotechnology and stimulus sensitive biomaterials for delivery of therapeutic compounds in a spatiotemporally controlled manner. Nanotechnologies represent a broad category of biotechnology which has been used to enhance cellular differentiation and promote bone formation both in vitro and in vivo [348]. Both organic and synthetic nanoparticles represent avenues for targeted drug delivery while also providing biomechanical signals for osteogenic differentiation [348]. These have been combined with bioorthogonal delivery systems to allow for coordinated release of therapies in a localized and controlled manner [349]. Demonstrating two in vivo use cases, Lee et al. and Sun et al. controlled the sequential release of rhBMP-2 through specialized HA-collagen or mesoporous BAG scaffolds in the setting of critical-sized bone defects to enhance bone healing in rat and canine models [103]. The continued evolution of molecular tools to directly target genes and their regulatory systems are another evolving field which may improve these interventions. These include the use of CRISPR-Cas9, CRISPR interference, and non-coding RNA systems to modulate gene therapy-based systems [350,351]. Finally, the integration of the aforementioned technologies to produce an optimal delivery device which enables the release of an exact milieu of key regulatory proteins remains a major goal. Park et al. leveraged several of these emerging concepts to improve bone tissue regeneration. They used CRISPR/Cas9 to overexpress BMP2 and VEGF in MSCs which were delivered in a multifunctional vitamin-D-incorporated magnesium hydroxide PLGA scaffold designed to optimally induce macrophage polarization and coordinated angio-osteogenesis with evidence of a robust healing response in vivo [352]. Despite early successes, given the complexity of bone healing in both the acute and chronic setting, integrated systems such as those employed by Park et al. are likely needed to produce optimal results. Further optimization of these therapies may be discovered with the use of machine learning (ML) algorithms; however, the utility of these advances have yet to be validated [353]. ML has also been used to identify patients at risk of nonunion using radiographic parameters, however in their current form, are unable to achieve the high levels of sensitivity or specificity required for widespread clinical adoption [354,355,356]. Moreover, while genetic, transcriptomic, and molecular signatures related to nonunion risk have been identified, their integration into a clinically useful tool has yet to be established, thus ML models will be a key approach for their optimization [357,358]. Finally, further large animal research is needed for translation of the pre-clinical data discussed herein, particularly given that rodents lack true Haversian cortical bone remodeling thus are not ideal models of bone repair [359]. In this context, regional gene therapy and scaffold-based delivery show some promise given their efficacy in large animal models in inducing enhanced bone repair; however, further efficacy and safety data are needed prior to clinical trials [96,98,360,361].

## 7. Concluding Remarks

Nonunion remains a complex orthopedic challenge with no gold standard treatment. Nonunion is highly prevalent and places a significant burden on patients and the healthcare system. Surgical intervention is the primary approach to treatment; however, these commonly require multiple surgeries and continue to have non-insignificant failure rates. Emerging treatments aim to replicate the ideal environment for bone healing with growing emphasis on restoring the functional and structural integrity of bone. Stem-cell-based therapies combined with gene therapy hold a promising future as they can be integrated with scaffolds and key growth factors for bone regeneration. Nonetheless, further research is needed to develop the optimal delivery system, cell type and gene targets to find an effective solution for nonunion.

## Figures and Tables

**Table 1:** Summary of recent key clinical and non-clinical studies related to fracture healing.

Study (Year)	Population/Model	Intervention	Comparator	N	Outcomes	Safety Notes	Reference
**Clinical Studies**							
Mazzotta et al. (2021)	Retrospective cohort of upper-limb aseptic nonunions	BMAC and PRF applied on lyophilized bone chip or bone graft following fixation	Fixation without addition of PRP/BMAC	45	Accelerated healing processes of lesions up to 6 cm in the upper limb	No adverse reaction reported	[89]
Laubach (2022)	Prospective pilot of large lower extremity bone defects	Patient specific 3D printed mPCL-TCP scaffolds with autologous bone graft	None	4	Three of the cases showed evidence of bone formation/bony fusion and were weight bearing within 9 months of scaffold placement. One case, which also included the use of BMP2, achieved bony fusion and underwent hardware removal	No adverse reaction reported	[90]
Xie (2022)	Meta-analysis of long bone nonunions	Autologous bone graft with rhBMP	Autologous bone graft alone	394	While the combination of ABG with rhBMP demonstrated better postoperative limb function compared to ABG alone, it did not result in improved healing times or postoperative pain.	No adverse reaction reported	[91]
Choi et al. (2024)	Prospective case series of long bone nonunion or bone defect	rhBMP-2 combined with autologous bone and hydroxyapatite carrier granules	None	24	All patients achieved union at 12 months	Reported that no adverse effects or development of BMP2 antibodies were observed.	[92]
Moyal (2024)	Systematic review of long bone nonunion or delayed union	Bone marrow aspirate (BM) and bone marrow aspirate concentrate (BMAC)	Scaffolds (porous collagen + bovine fibrillar collagen, demineralized bone matrix (DBM) or DBM composite, allogenic graft, bioactive glass, iliac bone autograft)	25 studies (encompassing approximately 580 patients)	BM and BMAC can lead to satisfactory union rats but the data is heterogeneous and higher quality studies are needed to understand BM/BMAC efficacy in relation to nonunion	Studies included 2 deep wound infection and 1 heterotopic ossification	[93]
Zhu (2024)	Umbrella meta-analysis of nonunion and delayed union	PRP	Any control group	5 meta-analyses (13 studies, 1362 patients)	As a whole, PRP used in the treatment of nonunion led to superior healing rates and improved healing time but when looking at individual studies PRP did not lead to improved healing rates but still led to improved healing time.	Adverse events found to be nonsignificant; Reported postoperative infections as adverse event following PRP	[94]
Tanavalee (2025)	Randomized controlled trial of patients >50 years old with pertrochanteric fractures undergoing surgical fixation	Teriparatide	Placebo	50	Teriparatide improved healing times of pertrochanteric fractures but did not lead to superior functional quality metrics (Harris hip score, time up and go test) or a significant difference in bone mineral density loss compared to the placebo.	No drug-related adverse events; Bruise at injection site at wk2/placebo group had skin itching around the injection area	[95]
**Selected Preclinical Studies**							
Pelled (2015)	Minipigs with lumbar vertebrae critical-size cylindrical bone defect, 15 mm in depth and 4 mm in diameter	Allogeneic BMP6 producing mesenchymal stem cells in fibrin gel	Fibrin only	6	Higher bone regeneration with increased connectivity density and bone volume on microCT analysis. Significance was not reached, likely due to N = 3 per group.	No adverse reactions reported	[96]
Brunello (2020)	Systematic review	Bioceramics	Empty defect	13 studies (6 in rats; 7 in rabbits)	Due to heterogeneity in protocols no meta-analysis could be performed. Higher healing proportion in treated defects	No adverse reactions reported	[97]
Liu (2021)	6 sheep with femoral or humeral 8 or 13 mm defects	Interior or surface BMP2 coated Biomimetic Calcium Phosphate (BioCaP) Granules	Empty defect; autologous bone graft; Demineralized bone graft; BioCaP alone	72 defects	Significantly greater bone volume and density in BMP2 BioCaP treated defects than empty defects, demineralized bone graft, and BioCaP alone; No differences between autologous or interior/surface BMP2 BioCP	No adverse reactions reported	[98]
DeBaun (2022)	8mm critical sized rat femoral defect	PCL/TCP scaffold enveloped by BMP2 or PDGF or BMP2+PDGF microspheres	Empty defect or PMMA spacer followed by PCL/TCP scaffold	40	Significantly higher radiographic healing scores in BMP2 treated subjects; No differences between BMP2 versus BMP2+PDGF	1/40 had a deep wound infection; No intervention-based safety concerns	[99]
De la Vega (2022)	5mm critical sized rat femoral defect	Chemically modified mRNA encoding for BMP2 implanted on a collagen sponge	Collagen sponge containing rhBMP2	190	cmRNA was faster as restoring mechanical strength and initiating bony bridging likely through endochondral ossification	No adverse reactions reported	[100]
da Rocha et al. (2023)	Paravertebral implantation in immunodeficient mice	Bone allograft with BMSC	β-TCP scaffold with and without BMSC; Bone allograft without BMSCs	12	Bone allograft with BMSC had significantly higher ALP activity and bone formation assessed via Alizarin red staining	No adverse reactions reported	[101]
Garot (2023)	25mm sheep metatarsal defect	3D printed polylactic acid scaffold with polyelectrolyte film coating delivering BMP2	Scaffold without BMP2	24	Cubic scaffolds with BMP2 (5/7 with full bridging) showed significantly improved bone formation than controls (0/4 with any bridging) and gyroid scaffold (3/7 with full bridging) on microCT with trends towards improved bone formation	No adverse reactions reported	[102]
Sun (2024)	5mm critical sized rat femoral defect and 2cm critical sized beagle radial defect	Mesoporous bioactive glass (MBG) with deforoxime-induced hypoxia-mimicking scaffold with BMP2 embedded PEGylated poly	BMP2 loaded MBG	12 rats; 12 beagles	Significantly higher vessel volume, bone volume percent, and bone mineral density with higher type I collagen versus type II collagen in the experimental rat group; Significantly higher bone volume percent (85% vs. 64%) and bone mineral density (1.3 vs. 0.9 g/cm3) in beagle group; Observed that BMP2 volume could be reduced to 1/10 using this system	High heterotopic ossification rates in the high (10 microgram) BMP2 dose groups versus low dose (1 microgram) (55% vs. 10%)	[103]
Li (2025)	5 mm cynomolgus monkey ulnar defect	Romosozumab, a sclerostin antibody	Control vehicle	22	New bone volume and new bone area within the defect region were 118% and 105% greater, respectively	No adverse reactions reported	[104]

**Table 2:** Summary of advantages and limitations of clinical and preclinical modalities used to promote fracture healing.

Intervention		Advantages	Limitations	References
**Clinical**				
External Bone Growth Stimulation		- Noninvasive	- No proven clinical benefit in healing nonunion	[107,108,142,143]
Bone grafting		- Can contain osteogenic and osteoinductive cells - Provides structural support and a scaffold for bone conduction	- Donor site morbidity - Limited availability	[144,145,146]
Autologous Orthobiologics		- Contains important growth factors for osteoinduction - Relative ease of acquisition with low donor site morbidity	- Cost and availability - Does not provide structural support - Inconsistent growth factor and cell counts across harvests	[93,147,148]
Recombinant Growth Factors		- Can be osteoinductive	- Difficulty in achieving local, therapeutic levels	[149,150]
**Preclinical**				
Scaffolds	Bioceramics	- Mimics the mechanotransductive properties of the ECM - Provides a foundation for bone conduction - Its micropores facilitate angiogenesis, cell adhesion, and bone deposition	- Brittle and fragile on their own - No inherent osteoinduction - Nonideal resorption kinetics	[151,152,153,154]
	Natural Polymers	- Resembles ECM - Low relative cost - Generally high biocompatibility and biodegradable	- Variability in release profiles of bioactive molecules, composition, and strength - Technological limitations in fabrication	[152,153,154,155,156]
	Hydrogels	- Can form to shape of defect site - Environment responsive - Elution dose and rates can be modulated to achieve therapeutic targets	- Do not provide structural support - Degradation products may not be biocompatible and induce a local tissue response	[152,153,154,157]
Stem cells		- Differentiate into osteogenic cells directly to promote union - Overall favorable safety profile with limited adverse events	- Cost of manufacturing and testing - Potential for immunogenicity with allogeneic cell therapy - Risk of oncogenesis - May require additional factors to promote union	[158,159,160,161]
*Gene therapy*		- Allows for sustained, local delivery of key osteoinductive proteins - Limited off target effects - Potential for synergistic multi-gene delivery to optimize healing	- Risk of immunogenicity and resulting impairment of efficacy - Risk of mutagenesis - High regulatory burden for clinical translation	[162,163,164,165]
*Combined therapy*		- Combine the advantages of each therapy modality and reduce the limitations	- Cost - Optimal dosing and delivery mechanisms remain poorly defined	[166,167]

**Table 3:** Summary of key non-BMP growth factors implicated in fracture healing.

Growth Factor	Mechanistic Target	Biologic Effects	References
VEGF	VEGF tyrosine kinase receptor; MAPK pathway; Endothelial cells	Angiogenesis; enhanced vascularization; improved graft survival	[171]
PDGF	PDGF tyrosine kinase receptor; MAPK pathway; Fibroblasts, smooth muscle cells, and glia cells	Fibroblast proliferation; wound healing; extracellular matrix production	[172]
PTH	PTH1 receptor (Gs/cAMP/PKA and PLC pathways); Osteoblasts and osteocytes	Intermittent dosing stimulates bone formation; improved callus strength; enhanced osteoblast differentiation and survival	[173,174,175,176,177]
DIPY	Adenosine receptor (A2A, A2B [ADORA2A/ADORA2B]) signaling; inhibits adenosine reuptake leading to osteoblast activation	Promotes osteogenesis; enhanced bone regeneration around scaffolds and implants; reduces osteoclast activity	[148,149,150,151,152,153,154,155,156,157,158,159,160,161,162,163,164,165,166,167,168,169,170,171,172,173,174,175,176,177,178,179,180]
FGF	(FGFR1–4); RAS/MAPK and STAT signaling pathways; Osteoblasts, chondrocytes, endothelial cells	Promotes angiogenesis and vascularization, osteogenesis through osteoblast differentiation; enhances chondrogenesis and endochondral ossification	[181]
TNF-alpha	TNFR1/TNFR2 (TNFRSF1A/TNFRSF1B) activates NF-kB and MAPK pathway; Immune regulation and MSC recruitment	Promotes early regulation of the initial inflammatory phase of fracture healing and MSC migration; Enhances osteoblast differentiation	[182]

**Table 4:** Overview of material properties of scaffold-based therapies.

Material	Pore Size (μm)	Strength (MPa)	Degradation Kinetics	Notes	Reference
Bone (cortical)	minimum >100	100–200	Not applicable	Ideal material properties of therapeutic scaffolds may differ from these ranges depending on mechanism of effect	[231]
Calcium-Phosphate ceramics	250–350	100–350	>95% at 90 days	Variability in measures dependent on composite scaffold design	[232,233]
Synthetic Polymers	Engineering allows for modifiable pore sizes	35–2300	variable, ranging from 1 to >24 months	PLA/PCL fibers much stronger with longer degradation times versus PLGA/PGA	[234,235]
Natural Polymers	50–400	0.05–60	variable, average range from 1 to 3 months	Silk fibroin and chitosan are stronger with longer degradation times versus hyaluronic acid, gelatin, aliginate, and collagen-based polymers	[236,237,238]
Hydrogels	0.01–100	10–90	days to ~8 weeks	Generally low structural support and longevity unless modified with embedded synthetic polymers	[236,237,239,240,241,242,243,244]

## Data Availability

Not applicable.
